# RETREG1/FAM134B-mediated micro-ER-phagy in the retrovirus–SERINC5 arms race

**DOI:** 10.1080/27694127.2025.2602971

**Published:** 2025-12-19

**Authors:** Jim Maurice Camilleri, Iqbal Ahmad, Jing Zhang, Sunan Li, Yong-Hui Zheng

**Affiliations:** a Department of Microbiology and Immunology, University of Illinois, Chicago, IL, USA; b State Key Laboratory for Animal Disease Control and Prevention, Harbin Veterinary Research Institute, Chinese Academy of Agricultural Sciences, Harbin, China

**Keywords:** Autophagy, ER-phagy, FAM134B, glycogag, micro-ER-phagy, restriction factor, reticulophagy, RETREG1, retrovirus, SERINC5

## Abstract

Reticulophagy regulator 1 (RETREG1)/Family with sequence similarity 134 member B (FAM134B) is a selective endoplasmic reticulum (ER)-phagy receptor that mediates starvation-induced macro-ER-phagy, but whether it participates in other pathways mediating ER turnover has remained unclear. Here, we unveil a previously unrecognized role for RETREG1 in micro-ER-phagy and show how the murine leukemia virus (MLV) accessory protein glycosylated group-specific antigen (glycoGag) exploits this pathway to antagonize the host restriction factor SERINC5 (serine incorporator 5). GlycoGag binds SERINC5 in the endoplasmic reticulum (ER) and selectively recruits RETREG1 to eliminate SERINC5 through an autophagosome-independent process that bypasses ATG3 (autophagy-related), ATG5, ATG7, BECN1 (Beclin-1), LC3 (microtubule-associated protein 1 light chain 3) lipidation, and PIK3C3 (phosphatidylinositol 3-kinase catalytic subunit type 3)/hVPS34 (vacuolar protein sorting 34). RETREG1 knockout abolishes degradation of ER-retained SERINC5, whereas endolysosomal turnover of surface SERINC5 remains partially intact, demonstrating that glycoGag utilizes dual ER-phagy and endolysosomal routes to suppress SERINC5. These findings expand the functional repertoire of RETREG1 in autophagy, identify that retroviruses repurpose micro-ER-phagy to circumvent SERINC5-mediated restriction, and reveal ER-phagy as an understudied battleground in the ongoing arms race between cellular restriction factors and viral accessory proteins.

Selective autophagy of the endoplasmic reticulum (ER-phagy/reticulophagy) is a key mechanism for maintaining ER proteostasis. Central to this process are the RETREG (reticulophagy regulator)/FAM134 (family with sequence similarity 134) family of ER-phagy receptors: RETREG1/FAM134B, RETREG2/FAM134A, and RETREG3/FAM134C. These receptors share an N-terminal reticulon-homology domain (RHD) that drives ER membrane remodeling and a C-terminal LC3-interacting region (LIR) that links ER fragments to the autophagy machinery. RETREG1, the first ER-phagy receptor identified, is best known for its role in starvation-induced macro-ER-phagy, where its RHD promotes membrane curvature and its LIR recruits LC3 to support autophagosome formation. Yet RETREG1 also participates in ER remodeling, collagen homeostasis, and ER stress responses, suggesting broader functions possibly beyond macro-ER-phagy (Figure 1).

**Figure 1.**
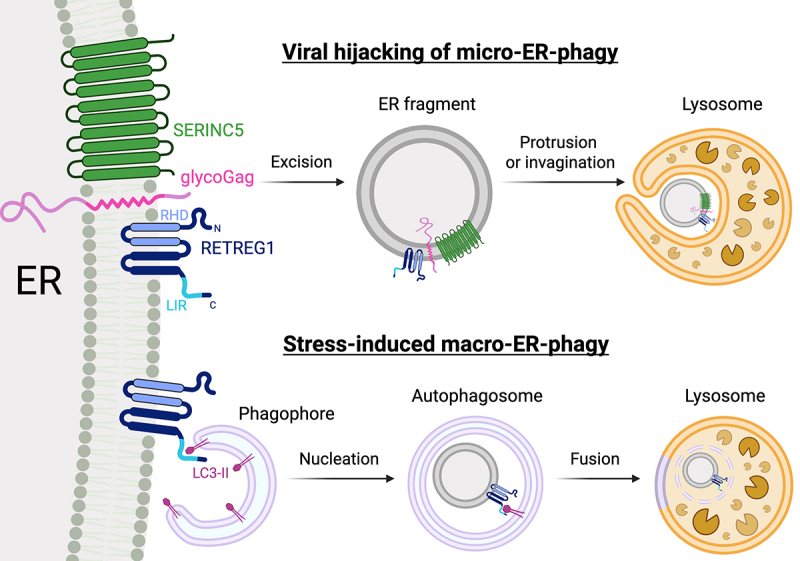
MLV glycoGag induces RETREG1-dependent micro-ER-phagy to antagonize SERINC5.

In our recent study^[1]^, we identify a previously uncharacterized function of RETREG1 in micro-ER-phagy and show how the murine leukemia virus (MLV) accessory protein glycoGag exploits this pathway to neutralize the host antiviral protein SERINC5 (serine incorporator 5). SERINC5 is a restriction factor that inhibits entry of multiple enveloped viruses. Retroviruses counteract SERINC5 using accessory proteins such as human immunodeficiency virus type 1 (HIV-1) Negative factor (Nef) and MLV glycosylated group-specific antigen (glycoGag). While both antagonists remove SERINC5 from the cell surface, our study reveals that glycoGag employs an additional mechanism, it repurposes the ER-phagy receptor RETREG1 to eliminate SERINC5 through micro-ER-phagy.

GlycoGag is an N-terminally extended, glycosylated isoform of the MLV Gag polyprotein. Although predicted to be a type II membrane protein, glycoGag predominantly appears in cytoplasmic puncta, unlike the plasma membrane-localized Nef. Cycloheximide-chase experiments show that glycoGag is highly unstable, with a half-life under 30 minutes, and is rapidly degraded by the proteasome. Its levels increase when the Cul3 (Cullin-3) – KLHL20 (Kelch-like protein 20) E3 ubiquitin ligase is inhibited, mirroring the behavior of other short-lived viral antagonists such as HIV-1 Vif (viral infectivity factor).

We show that glycoGag binds SERINC5 in the ER and stabilizes itself through this interaction. Strikingly, glycoGag selectively recruits RETREG1, but not RETREG2, RETREG3, or other selective ER-phagy receptors, to drive microautophagic engulfment of SERINC5-containing ER subdomains. This pathway operates independently of ATG3 (autophagy-related), ATG5, ATG7, BECN1 (Beclin-1), LC3 (microtubule-associated protein 1 light chain 3) lipidation, PIK3C3 (phosphatidylinositol 3-kinase catalytic subunit type 3)/hVPS34 (vacuolar protein sorting 34), and SQSTM1 (sequestosome-1)/p62, indicating that glycoGag activates an unconventional, autophagosome-independent form of ER-phagy. RETREG1 knockout completely blocks glycoGag-mediated degradation of ER-retained SERINC5, while degradation of cell-surface SERINC5 via delivery into endolysosomal compartments remains partially intact. Thus, glycoGag deploys two harmonizing mechanisms, RETREG1-mediated micro-ER-phagy and endolysosomal internalization, to efficiently suppress SERINC5 activity, surpassing the potency of HIV-1 Nef.

Macro-ER-phagy and micro-ER-phagy represent two distinct pathways that maintain ER homeostasis. Starvation and certain terminally misfolded host proteins activate macro-ER-phagy, in which the LC3-interacting region (LIR) of the ER-phagy receptor RETREG1 engages LC3-II on phagophore membranes. With the assistance of core autophagy-related factors, a *de novo* generated phagophore expands and seals to form autophagosomes that ultimately fuse with late endosomes and/or lysosomes. Micro-ER-phagy is less defined but is thought to involve transient protrusions or invaginations of lysosomal membranes that directly engulf ER-derived cargo. We show that MLV glycoGag co-opts RETREG1 to drive micro-ER-phagy, promoting the degradation of the host restriction factor SERINC5 and thereby evading its antiviral activity. This glycoGag-directed pathway is likely to depend on the reticulon-homology domain (RHD) of RETREG1.

The precise mechanism by which RETREG1 executes micro-ER-phagy remains unclear. Because its LIR is dispensable in our system, RHD-driven membrane remodeling may be sufficient to initiate lysosomal internalization, but whether this involves lysosomal invagination, protrusion, or ESCRT (endosomal sorting complex required for transport)-dependent engulfment is unknown. What host factor mediates the 3-MA-sensitive but LY294002-insensitive step required for SERINC5 degradation? This component, potentially a PI3K-related or PI3K-independent regulator, may define a unique signaling module for micro-ER-phagy. How widely do viruses exploit RETREG1 via micro-ER-phagy to promote their replication? Given that SERINC5 restricts many enveloped viruses, RETREG1-dependent micro-ER-phagy may represent a recurrent viral immune-evasion strategy. Finally, the physiological role of RETREG1-mediated micro-ER-phagy in uninfected cells needs to be further investigated, and we contemplate potential contributions to basal ER membrane turnover, protein quality control, and/or starvation-induced ER remodeling.

Together, our findings identify RETREG1 as a central node linking micro-ER-phagy to viral antagonism of host restriction factors, revealing new principles in selective autophagy and cell-autonomous immunity.

## Data Availability

Data sharing is not applicable to this article as no data were created or analyzed.
